# A comparison of drinking behavior using a harmonized methodology (*Liq.In*^*7*^) in six countries

**DOI:** 10.1007/s00394-018-1744-8

**Published:** 2018-06-12

**Authors:** C. Morin, J. Gandy, L. A. Moreno, S. A. Kavouras, H. Martinez, J. Salas-Salvadó, I. Guelinckx

**Affiliations:** 1 Department of Hydration and Health, Danone Research, Route Départemental 128, 91767 Palaiseau, France; 2 British Dietetic Association, Birmingham, UK; 3 School of Life Medical Services, University of Hertfordshire, Hatfield, UK; 4 GENUD (Growth, Exercise, NUtrition and Development) Research Group, Faculty of Health Sciences, Instituto Agroalimentario de Aragón (IA2), Instituto Investigación Sanitaria Aragón (IIS Aragón), Universidad de Zaragoza, Zaragoza, Spain; 5 CIBERobn (Centro de Investigación Biomédica en Red Fisiopatología de la Obesidad y Nutrición), Institute of Health Carlos III, Madrid, Spain; 6 Hydration Science Lab, University of Arkansas, Fayetteville, AR USA; 7 Division of Endocrinology, University of Arkansas for Medical Sciences, Little Rock, AR USA; 8 Hospital Infantil de México Federico Gómez, Mexico City, Mexico; 9 Biochemistry and Biotechnology Department, Human Nutrition Unit, Faculty of Medicine and Health Sciences, Hospital Universitari de Sant Joan de Reus, Institut d’Investigació Sanitària Pere Virgili, Universitat Rovira i Virgili, Reus, Spain

**Keywords:** Beverages, Fluid intake, Water, Hydration, *Liq.In*^*7*^, Behavior

## Abstract

**Purpose:**

To assess drinking occasions (volume and type) according to consumption with food in or outside meals, and location, for six countries.

**Methods:**

A total of 10,521 participants aged 4–65 years from Argentina, Brazil, China, Indonesia, Mexico and Uruguay completed a validated 7-day fluid intake record. For each drinking event, the volume consumed, the fluid type, the location of intake, and whether the drink was accompanied by food (meal or snack) or not, was recorded.

**Results:**

Similar drinking behaviors were found in Mexico and Argentina; fluid intake during meals was 48 and 45% of total fluid intake (TFI), respectively. In Brazil (55%), Indonesia (58%) and China (66%) most fluid was consumed without food. In Uruguay, 34% of TFI was with a main meal, 31% with food between meals and 35% without food. Indonesia had the highest median (25–75th percentile) TFI; 2520 (1750–3347) mL/day, and China the lowest 1138 (818–3347) mL/day. Water was consumed with meals for 37% of Chinese and 87% of Indonesian participants, while the four Latin-American American countries showed a preference for sweet drinks; 54% in Mexico, 67% in Brazil, 55% in Argentina and 59% in Uruguay. Diversity in fluid type was noted when drinking with food between meals. Apart from China, most drinking occasions (> 75%) occurred at home.

**Conclusions:**

Three distinct drinking behaviors were identified, namely, drinking with meals, drinking as a stand-alone activity, and a type of ‘grazing’ (i.e., frequent drinks throughout the day) behavior. Most drinking occasions occurred at home.

**Electronic supplementary material:**

The online version of this article (10.1007/s00394-018-1744-8) contains supplementary material, which is available to authorized users.

## Introduction

Recent interest in the effects of hydration on health and disease [1–4] has resulted in increased reporting of total water intake (water from food moisture, drinking water and all other fluids) or total fluid intake (TFI) in many populations around the world [5–8]. These publications have identified countries or subpopulations potentially at risk of health consequences related to hypohydration. As a result, behavior change programs that encourage consumption, particularly of healthy options, have been gaining attention. Ideally these programs should be designed to target the location and circumstances of consumption that will have the most impact. However, drinking behavior needs to be better understood in order to make behavior change in a particular setting (e.g., at home, in schools or the workplace). To facilitate a better understanding of drinking behavior, it is now apposite to study drinking behaviors in terms of not only what is drunk, but also when (e.g., with or without food) and where.

Increasingly, food and drink are being consumed outside the home. For example, in the USA, expenditure on food away from home increased from nearly 26% of total expenditure in 1970 to 43% in 2012 [9]. This change in behavior is being echoed in other, less affluent, countries such as Brazil [10]. This is perhaps unsurprising given the increasing amount of time spent away from home with increasing leisure time in many countries, particularly developed countries such as the UK [11]. Eating in food outlets and “on the go” (food consumed away from a table and usually outside) has been associated with a less healthy diet [12]. A study by Nissensohn et al. [13] is one of the few that has attempted to look at drinking behavior and relate this to a variety score that in turn relates to health.

Drinking behavior, like eating behavior, is influenced by many factors including culture, religion, familial and peer influences, socioeconomic status, geographic location, taste preferences, etc. [14–16]. Research on drinking habits and the location of drinking occasions is an emerging area of interest; however, most studies focus on energy-containing drinks, especially sugar-sweetened beverages (SSB) [12, 17–20]. Undoubtedly, more research is needed on this topic. Therefore, it is important to consider the most appropriate methodology that will capture all drinking occasions throughout the day and will also describe drinking habits [21]. The chosen methodology must be robust and able to capture an accurate picture of drinking behavior beyond 1 day, as it has been documented that drinking behavior changes over the course of a week [22]. There is increasing research into fluid consumption during the day [13, 18, 21, 23–25] and over the week [26–28] although most methodologies have inherent limitations when recording fluid intake. The use of a more appropriate methodology to study drinking behavior should further the understanding of this behavior.

While some studies, particularly national diet and nutrition surveys, e.g., Kerr et al. [29], report consumption of fluids and foods, none have looked at drinking habits in relation to whether or not food was consumed with the fluid. Therefore, the primary aim of this study was to describe fluid intake during meals, other eating occasions outside of meals, and stand-alone drinking occasions (i.e., without food). The secondary aim of the present study was to identify the location of the drinking occasion.

## Methods

### Study population

The recruitment of participants and further details of the populations included in this analysis have been described previously [30–33].

### Assessment of total fluid intake and fluid types

Participants were provided with the *Liq.In*^*7*^ record; a 7-day fluid-specific record validated for accuracy and reliability [34]. The *Liq.In*^*7*^ record was presented in the official country language. The record had the same structure and content in all countries; this was adapted according to the brands available in each country. The record was delivered and explained to the participants during an interview at home. After a period of 7 days, the paper record was collected by the researcher and checked with the participant for completion. An electronic version of the record was used in China. The *Liq.In*^*7*^ record was structured according to occasions during the day, namely, awakening, meal times (breakfast, lunch, dinner), periods between meals (morning, before lunch/aperitif, afternoon, tea break, before dinner/aperitif, evening, just before going to bed) and during the night. The participants were instructed to report all drinking events at any moment of the day with the following details: fluid type, size of the container from which the fluid was drunk, actual volume consumed, where the consumption took place and if the fluid was consumed with or without food. *Liq.In*^*7*^ does not record food consumption. To assist the participants in estimating the precise volume of fluid consumed, a photographic booklet of standard fluid containers supported the records. For children younger than 12 years, the primary caregiver was responsible for completing the record.

### Classification and analysis of fluid types

Characteristics of TFI and consumption of different fluid types in the six countries are discussed further in other articles [30–33]. The fluids recorded were classified as: water (tap and bottled water); milk and milk derivatives; hot beverages (coffee, tea and other hot beverages); 100% fruit juices; sugar-sweetened beverages (SSB) being carbonated soft drinks (CSD), juice-based drinks, functional beverages, e.g., energy and sports drinks, ready-to-drink tea & coffee and flavored water; artificial/non-nutritive sweeteners beverages (A/NSB) (diet/zero/light soft drinks); and other beverages. Volumes of all categories were summed to give total fluid intake (TFI).

### Ethical considerations

Participants were given detailed information about the survey’s objectives, their involvement, their rights to confidentiality, risks and benefits, and a clear explanation that participation in the survey was entirely voluntary. All participants gave informed oral consent and no monetary incentive was offered to take part in the survey. All data were recorded and analyzed anonymously. The survey protocol was reviewed and approved by the University of Arkansas Review Board (ref. 14-12-376).

### Statistical analysis

Participants who did not complete the full 7 days of the *Liq.In*^*7*^ record, those who reported a mean total daily fluid intake < 400 or > 4000 mL/day for children younger than 14 years and > 6000 mL/day for participants older than 14 years were excluded from the analysis. Due to the skewed distribution in intakes, TFI per drinking occasions and location are presented as medians and 25–75th percentiles as well as mean and standard error of mean. The intakes of the different fluid types are reported as median (25–75th percentiles). The mean and standard error of mean (SEM) of the fluid types can be found in the Online Source Tables S1a–c. As there were limited and inconsistent gender differences, these data are not presented according to gender.

The drinking occasions were classified into three categories (1) “meals” meaning that the act of consumption was during a main meal, (2) “outside of meals” meaning that the act of consumption was taken with food but not during one of the main meals, and (3) “without food” meaning that the act of consumption was taken without any food (a stand-alone drinking occasion).

Locations of consumption were categorized for analysis into the following categories; at home, at school/work/university, including cafeterias, and all other locations, e.g., restaurant/bar/public house, transportation, friend/acquaintance’s house, sports venue, shopping center, street, park, hotel, hospital. The variable “location” was not completed for all fluid intake acts, and these are reported in the online resources as “Unspecified”.

## Results

### Study population

The demographic characteristics of study population aged 4–70 years (total sample size 10,521) for each of the six countries are shown in Table 1. Population characteristics per country and age group are shown in the Online Source Table S1.

**Table 1:** Demographic characteristics of the study population, by country

Country	Sample size	Gender		Age (years)
		Male	Female	
Mexico	2346	1098 (47)	1248 (53)	30 ± 17
Brazil	817	354 (43)	463 (57)	27 ± 18
Argentina	1481	708 (48)	773 (52)	31 ± 17
Uruguay	819	409 (50)	410 (50)	29 ± 17
China	2233	1120 (50)	1113 (50)	27 ± 14
Indonesia	3644	1778 (49)	1866 (51)	30 ± 15

Age reported as mean ± standard deviation and gender as number (percentage of country sample)

### Fluid intake according to drinking occasion

Table 2 shows the volume and contribution of TFI according to occasions for the total population in each country. Data and figures for individual age groups are given in the online resource Table S3 and Figure S1. Mexico and Argentina had broadly similar drinking behaviors: participants mainly drank during meals (48 and 45% of the TFI, respectively). However, for Brazilian (55%), Indonesian (58%) and Chinese (66%) participants drinking is most often a stand-alone activity, outside of meals without any food. Only a few Chinese participants reported eating and drinking together between meals (6%). The participants in Uruguay reported drinking throughout the day consuming 34% of TFI with a main meal, 31% with food between main meals and 35% without food, respectively.

**Table 2:** Daily total fluid intake (mL/day) according to country and drinking occasion and the contribution to total fluid intake

Country	Occasions	Mean	SEM	Median	P25	P75	Contribution to TFI (%)
Mexico (*n* = 2346)	TFI	1677	20	1431	999	2068	100
	Meals	810	12	708	402	1103	48
	Outside of meals	232	7	100	0	328	14
	Without food	636	14	441	171	886	38
Brazil^a^ (*n* = 817)	TFI	1723	33	1499	1060	2211	100
	Meals	474	13	397	201	657	28
	Outside of meals	224	10	143	48	301	13
	Without food	953	23	796	508	1218	55
Argentina (*n* = 1481)	TFI	2162	26	2022	1454	2715	100
	Meals	972	14	888	553	1312	45
	Outside of meals	493	11	429	164	720	23
	Without food	697	17	529	242	969	32
Uruguay (*n* = 819)	TFI	1895	33	1731	1210	2415	100
	Meals	653	16	584	323	867	34
	Outside of meals	579	21	427	143	806	31
	Without food	664	23	463	169	954	35
China (*n* = 2233)	TFI	1300	15	1138	818	1582	100
	Meals	370	6	300	173	481	28
	Outside of meals	73	4	0	0	72	6
	Without food	857	12	741	480	1113	66
Indonesia (*n* = 3644)	TFI	2631	19	2520	1750	3347	100
	Meals	684	8	630	376	900	26
	Outside of meals	426	8	291	68	600	16
	Without food	1521	15	1389	804	2106	58

*SEM* standard error of the mean, *P25* 25th percentile, *P75* 75th percentile, *TFI* total fluid intake

^a^“Unspecified” modality of variable not presented

### Fluid types according to drinking occasion

**Fig. 1:**
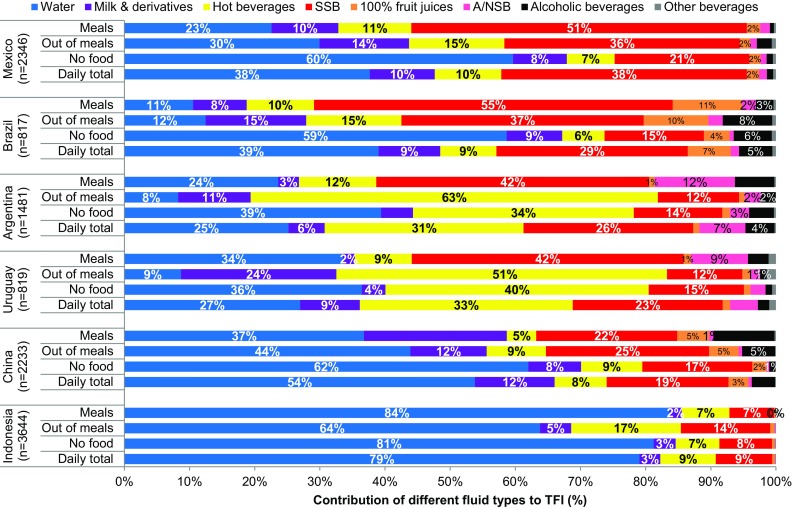
Contribution of the different fluid types to total fluid intake according to drinking occasion, by country

Table 3 and Fig. 1 show the median intakes of different fluid types and contribution to TFI by occasion respectively. These data by age group are presented in Figure S1. During main meals, sweet drinks (SSBs, A/NSBs and 100% fruit juices) were favored by the four Latin America countries; 54% of drinks in Mexico, 67% in Brazil, 55% in Argentina and 59% in Uruguay. These sweet drinks constituted only 28% of drinks consumed during meals in China and 7% in Indonesia. Water was favored during main meals in both China (37%) and Indonesia (84%).

**Table 3:** Median (25–75th percentiles) intake of fluid types (mL/day) according to drinking occasions

Country	Occasion	Water	Milk and derivatives	Hot beverages	SSB	100% fruit juices	A/NSB	Alcoholic beverages	Other beverages
Mexico (*n* = 2346)	Daily total	410 (166–846)	86 (0–257)	71 (0–243)	504 (275–863)	0 (0–0)	0 (0–0)	0 (0–0)	0 (0–0)
	Meals	57 (0–215)	0 (0–107)	0 (0–107)	326 (129–577)	0 (0–0)	0 (0–0)	0 (0–0)	0 (0–0)
	Outside of meals	0 (0–51)	0 (0–9)	0 (0–0)	0 (0–92)	0 (0–0)	0 (0–0)	0 (0–0)	0 (0–0)
	Without food	184 (9–516)	0 (0–50)	0 (0–34)	43 (0–171)	0 (0–0)	0 (0–0)	0 (0–0)	0 (0–0)
Brazil^a^ (*n* = 817)	Daily total	521 (304–858)	83 (0–247)	86 (0–221)	429 (216–677)	50 (0–163)	0 (0–0)	0 (0–51)	0 (0–0)
	Meals	0 (0–54)	0 (0–39)	0 (0–62)	189 (65–392)	0 (0–70)	0 (0–0)	0 (0–0)	0 (0–0)
	Outside of meals	0 (0–18)	0 (0–36)	0 (0–36)	34 (0–109)	0 (0–4)	0 (0–0)	0 (0–0)	0 (0–0)
	Without food	427 (234–728)	27 (0–107)	11 (0–71)	81 (12–192)	0 (0–43)	0 (0–0)	0 (0–0)	0 (0–0)
Argentina (*n* = 1481)	Daily total	350 (95–779)	0 (0–163)	536 (234–958)	411 (114–824)	0 (0–0)	0 (0–143)	0 (0–101)	0 (0–0)
	Meals	54 (0–350)	0 (0–0)	0 (0–143)	291 (64–632)	0 (0–0)	0 (0–101)	0 (0–37)	0 (0–0)
	Outside of meals	0 (0–32)	0 (0–36)	186 (0–489)	0 (0–64)	0 (0–0)	0 (0–0)	0 (0–0)	0 (0–0)
	Without food	136 (0–386)	0 (0–0)	75 (0–321)	0 (0–109)	0 (0–0)	0 (0–0)	0 (0–0)	0 (0–0)
Uruguay (*n* = 819)	Daily total	375 (150–736)	36 (0–286)	286 (0–1040)	300 (86–661)	0 (0–0)	0 (0–34)	0 (0–0)	0 (0–0)
	Meals	51 (0–357)	0 (0–0)	0 (0–0)	150 (0–409)	0 (0–0)	0 (0–0)	0 (0–0)	0 (0–0)
	Outside of meals	0 (0–25)	0 (0–207)	0 (0–340)	0 (0–50)	0 (0–0)	0 (0–0)	0 (0–0)	0 (0–0)
	Without food	109 (0–336)	0 (0–0)	0 (0–321)	0 (0–100)	0 (0–0)	0 (0–0)	0 (0–0)	0 (0–0)
China (*n* = 2233)	Daily total	554 (323–889)	129 (41–231)	13 (0–90)	172 (51–357)	0 (0–54)	0 (0–0)	0 (0–2)	0 (0–0)
	Meals	71 (4–179)	60 (0–129)	0 (0–0)	36 (0–120)	0 (0–0)	0 (0–0)	0 (0–0)	0 (0–0)
	Outside of meals	0 (0–18)	0 (0–0)	0 (0–0)	0 (0–0)	0 (0–0)	0 (0–0)	0 (0–0)	0 (0–0)
	Without food	410 (223–700)	34 (0–103)	0 (0–54)	86 (0–207)	0 (0–17)	0 (0–0)	0 (0–0)	0 (0–0)
Indonesia (*n* = 3644)	Daily total	1924 (1296–2707)	0 (0–80)	132 (0–333)	93 (0–311)	0 (0–0)	0 (0–0)	ND	0 (0–0)
	Meals	493 (245–773)	0 (0–0)	0 (0–36)	0 (0–34)	0 (0–0)	0 (0–0)	ND	0 (0–0)
	Outside of meals	135 (0–377)	0 (0–0)	0 (0–75)	0 (0–51)	0 (0–0)	0 (0–0)	ND	0 (0–0)
	Without food	1101 (585–1749)	0 (0–29)	0 (0–137)	17 (0–167)	0 (0–0)	0 (0–0)	ND	0 (0–0)

*SSB* sugar-sweetened beverages, *A****/****NSB* artificial/non-nutritive sweeteners beverages, *TFI* total fluid intake, *ND* no data

^a^Modality “Unspecified” of variable not presented

There was more diversity in the fluid types consumed with food outside of meals. In Mexico and Brazil sweet drinks (SSBs, 100% fruit juice and A/NSBs) remained the most popular drinks when eating food outside of meals (39 and 49%, respectively). In Argentina and Uruguay participants most frequently drank hot beverages (63 and 51%, respectively) on these occasions. Water was the most popular drink taken with food outside of meals in China (44%) and Indonesia (64%); however, 25% of fluid intake at these occasions in China was SSBs.

The most popular stand-alone drink (without food) was water with median (25th–75th percentiles) intakes ranging from 109 (0–336) mL/day in Uruguay to 1101 (585–1749 mL/day) in Indonesia. However, when expressed as percentages, water constituted 36% of fluid intake consumed without food compared with 40% for hot beverages. Data for each age category, by country, are shown in the Online Source Tables S4a–c.

### Location of fluid consumption

In all countries except in China, most drinking occasions (over 75%) occur in the home (Table 4). In China, the median (25th–75th percentiles) of fluid consumed at home was 476 (271–734) mL/day with 349 (174–601) mL/day being consumed at school, university or work; therefore, only 43% of TFI was consumed at home. The Online Source Table S5 and Figure S3 show median intakes by location and age group, by country and Tables S6 shows median intakes by location and age group, by country.

**Table 4:** Daily fluid intake (mL/day) by location, by country

Country	Location	Mean	SEM	Median	P25	P75	Contribution to TFI (%)
Mexico (*n* = 2346)	TFI	1677	20	1431	999	2068	100
	At home	1368	17	1170	800	1725	82
	At school/univ/work	177	8	0	0	184	11
	Other locations	132	6	0	0	143	8
Brazil^a^ (*n* = 817)	TFI	1723	33	1499	1060	2211	100
	At home	1323	27	1141	794	1672	77
	At school/univ/work	167	12	32	0	205	10
	Other locations	221	12	100	0	286	13
Argentina (*n* = 1481)	TFI	2162	26	2022	1454	2715	100
	At home	1646	21	1535	1057	2086	76
	At school/univ/work	339	13	154	0	507	16
	Other locations	177	8	25	0	254	8
Uruguay (*n* = 819)	TFI	1895	33	1731	1210	2415	100
	At home	1511	28	1405	917	1945	80
	At school/univ/work	289	16	114	0	350	15
	Other locations	95	10	0	0	16	5
China (*n* = 2233)	TFI	1300	15	1138	818	1582	100
	At home	560	18	476	271	734	43
	At school/univ/work	444	9	349	174	602	34
	Other locations	296	7	204	70	426	23
Indonesia (*n* = 3644)	TFI	2631	19	2520	1750	3347	100
	At home	2287	18	2133	1467	2906	87
	At school/univ/work	271	8	0	0	386	10
	Other locations	73	4	0	0	34	3

*SEM* standard error of the mean, *P25* 25th percentile, *P75* 75th percentile, *TFI* total fluid intake, *Univ* university

^a^Modality ^“^Unspecified” of variable not presented

**Fig. 2:**
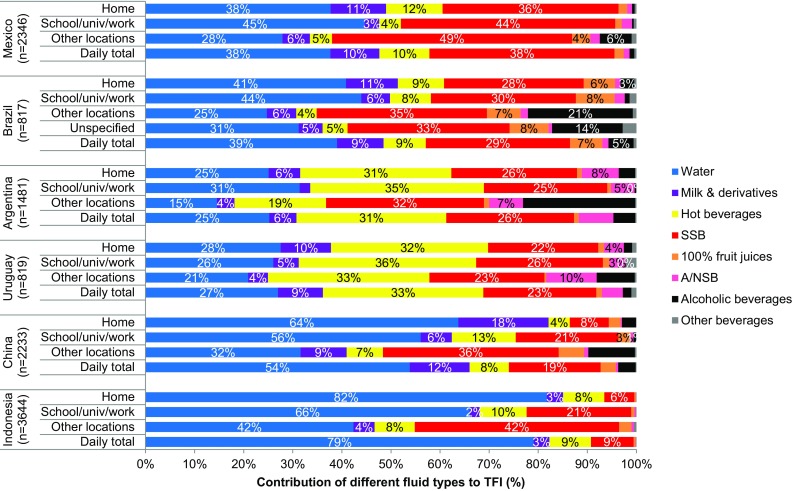
Contribution of the different fluid types to total fluid intake according to drinking location, by country

Table 5 and Fig. 2 show the median intakes of different fluid types and contribution to TFI by location respectively. In all countries the contributions of SSB and alcoholic beverages to TFI were higher in locations other than those at home or school, university or work. In China the contribution of hot beverages at work was higher than the one at home, while in Mexico the opposite was observed. In the other countries the contribution of hot beverages was comparable between the locations. In Indonesia the contribution of SSB to TFI at home was limited (6%), whereas it increased up to 21% at school, university or work and even 42% at other locations.

**Table 5:** Median (25–75th percentiles) intake of fluid types (mL/day) according to drinking locations

Country	Location	Water	Milk and derivatives	Hot beverages	SSB	100% fruit juices	A/NSB	Alcoholic beverages	Other beverages
Mexico (*n* = 2346)	Daily total	410 (166–846)	86 (0–257)	71 (0–243)	504 (275–863)	0 (0–0)	0 (0–0)	0 (0–0)	0 (0–0)
	Home	323 (103–707)	75 (0–231)	64 (0–214)	386 (180–673)	0 (0–0)	0 (0–0)	0 (0–0)	0 (0–0)
	School/univ/office	0 (0–0)	0 (0–0)	0 (0–0)	0 (0–43)	0 (0–0)	0 (0–0)	0 (0–0)	0 (0–0)
	Other locations	0 (0–0)	0 (0–0)	0 (0–0)	0 (0–63)	0 (0–0)	0 (0–0)	0 (0–0)	0 (0–0)
Brazil^a^ (*n* = 817)	Daily total	521 (304–858)	83 (0–247)	86 (0–221)	429 (216–677)	50 (0–163)	0 (0–0)	0 (0–51)	0 (0–0)
	Home	407 (233–681)	64 (0–206)	66 (0–180)	300 (136–511)	34 (0–107)	0 (0–0)	0 (0–0)	0 (0–0)
	School/univ/office	0 (0–79)	0 (0–0)	0 (0–0)	0 (0–50)	0 (0–0)	0 (0–0)	0 (0–0)	0 (0–0)
	Other locations	0 (0–43)	0 (0–0)	0 (0–0)	27 (0–105)	0 (0–0)	0 (0–0)	0 (0–0)	0 (0–0)
Argentina (*n* = 1481)	Daily total	350 (95–779)	0 (0–163)	536 (234–958)	411 (114–824)	0 (0–0)	0 (0–143)	0 (0–101)	0 (0–0)
	Home	250 (43–600)	0 (0–139)	401 (150–736)	278 (42–645)	0 (0–0)	0 (0–100)	0 (0–3)	0 (0–0)
	School/univ/office	À (0–86)	0 (0–0)	0 (0–129)	0 (0–86)	0 (0–0)	0 (0–0)	0 (0–0)	0 (0–0)
	Other locations	0 (0–0)	0 (0–0)	0 (0–0)	0 (0–54)	0 (0–0)	0 (0–0)	0 (0–0)	0 (0–0)
Uruguay (*n* = 819)	Daily total	375 (150–736)	36 (0–286)	286 (0–1040)	300 (86–661)	0 (0–0)	0 (0–34)	0 (0–0)	0 (0–0)
	Home	300 (86–643)	0 (0–250)	150 (0–817)	212 (11–536)	0 (0–0)	0 (0–0)	0 (0–0)	0 (0–0)
	School/univ/office	0 (0–25)	0 (0–0)	0 (0–0)	0 (0–0)	0 (0–0)	0 (0–0)	0 (0–0)	0 (0–0)
	Other locations	0 (0–0)	0 (0–0)	0 (0–0)	0 (0–0)	0 (0–0)	0 (0–0)	0 (0–0)	0 (0–0)
China (*n* = 2233)	Daily total	554 (323–889)	129 (41–231)	13 (0–90)	172 (51–357)	0 (0–54)	0 (0–0)	0 (0–2)	0 (0–0)
	Home	256 (107–477)	64 (0–157)	0 (0–0)	0 (0–64)	0 (0–0)	0 (0–0)	0 (0–0)	0 (0–0)
	School/univ/office	152 (43–334)	0 (0–36)	0 (0–36)	43 (0–135)	0 (0–0)	0 (0–0)	0 (0–0)	0 (0–0)
	Other locations	42 (0–118)	0 (0–38)	0 (0–18)	54 (0–150)	0 (0–0)	0 (0–0)	0 (0–0)	0 (0–0)
Indonesia (*n* = 3644)	Daily total	1924 (1296–2707)	0 (0–80)	132 (0–333)	93 (0–311)	0 (0–0)	0 (0–0)	ND	0 (0–0)
	Home	1714 (1127–2448)	0 (0–64)	103 (0–287)	34 (0–187)	0 (0–0)	0 (0–0)	ND	0 (0–0)
	School/univ/office	0 (0–196)	0 (0–0)	0 (0–0)	0 (0–0)	0 (0–0)	0 (0–0)	ND	0 (0–0)
	Other locations	0 (0–0)	0 (0–0)	0 (0–0)	0 (0–0)	0 (0–0)	0 (0–0)	ND	0 (0–0)

*SSB* sugar-sweetened beverages, *A****/****NSB* artificial/non-nutritive sweeteners beverages, *TFI* total fluid intake, *ND* no data

^a^Modality “Unspecified” of variable not presented

## Discussion

This study is the first time that drinking behavior, in terms of volume and fluid type, has been reported for these populations by including whether or not food was also consumed, either as a meal or outside of meals. We describe three distinct drinking behaviors: drinking with meals, drinking as a stand-alone activity and a type of ‘grazing’ (i.e., frequent drinking occasions throughout the day) behavior, which appear to be linked to social, cultural and dietary factors. This hypothetical link is based on the clear differences observed between countries. No comparable studies are available for Argentina, Brazil, Mexico, Uruguay or Indonesia, although there have been reports on drinking occasions from China. A study in schools in China showed that 71% of TFI are consumed outside meals [35], which is remarkably similar to the present study reporting 72%. However, Zhang [23] reported that 52% of the fluid drunk during meals was water compared with 37% in the present study. Water and fluid intake in China were quite different compared to the other countries in this analysis. The usual Chinese diet contains many dishes with a high water content (e.g., soups [36]); consequently, there is less need to drink fluid in order to chew and swallow food. The amount of total water intake derived from food in China has been estimated to be 40% [36] compared to 21% in Indonesia [37]. Data on the water provided by food in the diet of the Latin American countries included in this analysis were not available.

Chinese, Indonesian and Brazilian participants most frequently consumed fluids without food, while Mexican and Argentinian participants favored drinking with food, both during and between meals. The participants from Uruguay drank throughout the day, a behavior that may be described as ‘grazing’. Mate, a traditional hot infusion of the herb Ilex paraguayensis, is popular in Uruguay [31] and is consumed throughout the day, which may partly explain this behavior. Several studies have described drinking behavior during and between mealtimes [13, 25, 27, 38] and others have described drinking occasions across the day [23, 39]. As in the present study, differences between countries were observed: e.g., in France drinking is concentrated during meal times [25, 27, 40], whilst a Spanish study concluded that time of day had no effect [13] on drinking behavior. Social and cultural factors, such as purchasing resources, and environmental and fiscal conditions may have a role in determining the type of drinking habits in a particular country. However, this requires further study. In addition, more information is needed to be able to establish the importance of such habits and their relevance to health.

Water was the preferred drink when no food was eaten in all countries included in this study, except for Uruguay, which favored hot beverages, probably mate. However, there was more variation in the preferred type of drink when eating between meals. Eating food appeared to be a major determinant of fluid type choice especially during meals. To the best of our knowledge, only two other studies (both in children aged 4–17 years) have described the type of beverages according to meals and between meal occasions. A study of British children aged 4–13 years showed that 60% of fluids were consumed at meal times and that the drink of choice varied over the course of the day [38]. At breakfast the favored drinks were milk, 100% fruit juices and hot beverages; water-based fruit drinks (not 100% fruit juice) were favored at lunch, and fruit drinks, water, soda and milk at dinner time. Most SSBs were drunk at dinner time and in the afternoon. In contrast, a study in French children [40] showed that drinks were more likely to be consumed during meals than with the British children. Again, there was a variation in types of drink consumed across the day; milk was favored at breakfast, while water was favored at lunch and dinner. The consumption of SSBs was relatively low in both groups of children. It is difficult to make comparisons between the present study and these two aforementioned studies for many reasons including the differences in age groups studied, i.e., 4–13 years vs. populations that included children, adolescents and adults. Secondly, timings of meals and between meal periods were not recorded in the present study as the focus was on whether or not food was consumed at the drinking occasions at all. Conversely, whether or not food was consumed at a drinking occasion was not recorded in these former studies.

It is interesting to note that terminology has an impact on whether or not fluids are included in studies. For example, definitions of an eating occasion, a meal or, in particular, a snack vary and are often based on the energy contents of the snacks [41]. This may result in stand-alone drinking occasions not being recorded accurately, especially those in which energy is not consumed, e.g., plain water [41]. In the present study, drinking occasions when food was not consumed were variable, accounting for 32–55% of median fluid intake in the Latin American countries, 58% in Indonesia and 66% in China. These stand-alone occasions represent significant contributions to TFI, and energy intake however, they may have been ignored in some studies as no food was consumed [42]. Snacking has been shown to be increasing in many countries [21, 43, 44] and soft and carbonated drinks have been found to be the most popular drink/snack-combinations in some countries [45]. Encouragingly, some studies have recognized the importance of including drinks in such surveys [43, 45]. It is now pertinent to revise the definitions associated with the study of eating habits, particularly the definition of a snack, to accurately include drinking occasions regardless of energy content.

Most drinking occasions occurred at home for all of the Latin-American countries and Indonesia; in China less than half of the TFI was consumed at home. Similarly, it was found that for French children [40] most drinking occurred outside the home. The previously mentioned study of British children [38] found that most drinking occasions occurred at home although the greatest volume of fluid was consumed outside the home. The largest volume was consumed in full service restaurants followed by fast food restaurants; soda (regular and diet) was the preferred drink in both types of restaurant. This is in accordance with other studies, despite plain water often being available free-of-charge at full-service restaurants [17]. In the present study the contribution of SSB was largest at locations away from home, which contrasts with other studies that have found that most energy-dense beverages were consumed at home [20, 41]. Other studies [38, 40] have shown that water is consumed in the largest volumes in schools; however, these studies were conducted in countries that have legislation on what types of drinks are available in schools. Interestingly, [19] concluded that schools were a limited source of energy-dense beverages, especially when policies were in place to reduce their availability [46, 47]. However, these, and most other studies on this topic, have been conducted in USA, unlike the present intercontinental study. The influence of cultural and societal factors in drinking behavior requires further study. Encouragement of healthier drinking options, especially water, in schools and child care settings [48] will aid the development of healthy drinking behavior and facilitate education on this topic.

The present study has several strengths, not least the use of a validated methodology that captures all drinking occasions [34]. In addition, a harmonized survey methodology was used across all the studied countries that resulted in a population size of over 10,000 participants, which further strengthens the findings. The approach to categorize drinking occasions as to whether or not food was consumed, was innovative and facilitated interesting comparisons of cross cultural drinking behaviors. However, the sample size was insufficient to ensure a powerful analysis of fluid types according to locations. As with any form of dietary survey there is a potential for a selection bias, with people more interested in the research participating in the survey. In addition other factors that influence drinking behavior such as climate, level of education and physical activity have not been considered in this analysis.

## Conclusions

In conclusion, this is the first study to report drinking behavior in relation to eating occasions and location using harmonized and validated methodology. This study showed clear differences between countries and identified distinct drinking behaviors. These behaviors suggest that eating food was associated with the choice of fluid type. Further studies are needed to explore reasons for differences in drinking behavior, especially cultural factors. Understanding drinking habits is particularly important given the increasing recognition of the role of healthy hydration in the prevention and management of several diseases, including cardiometabolic and renal conditions. In particular, understanding drinking habits in terms of location should inform the rationale for further public health programs and policies. As such, culturally, and country-specific interventions will be more relevant to the targeted population and, therefore, hopefully more effective.

## Electronic supplementary material

Below is the link to the electronic supplementary material.

Supplementary material 1 (DOCX 239 KB)
