# Supplementary material for: A comparison of drinking behavior using a harmonized methodology (Liq.In7) in six countries
Source: Eur J Nutr. 2018 Jun 12;57(Suppl 3):101–12. doi: 10.1007/s00394-018-1744-8 (PMC6008358; doi:10.1007/s00394-018-1744-8)
Supplement: Supplementary file 1 — Supplementary material 1 (DOCX 239 KB) [file 394_2018_1744_MOESM1_ESM.docx]

**A cross-cultural comparison of drinking behavior using a harmonized methodology (*Liq.In^7^*) in 6 countries**

*Morin C,^1^ Gandy J,^2,3^ Moreno LA, ^4,5^ Kavouras SA,^6,7^ Martinez H,^8,9^ Salas-Salvadó J ^5,10^ Guelinckx I,^1*^*

^1^ Department of Hydration & Health, Danone Nutricia Research, Palaiseau, France

^2^ British Dietetic Association, Birmingham, UK

^3^ School of Life Medical services, University of Hertfordshire, Hatfield, UK

^4^ GENUD (Growth, Exercise, NUtrition and Development) Research Group, Faculty of Health Sciences, Universidad de Zaragoza, Instituto Agroalimentario de Aragón (IA2), Instituto Investigación Sanitaria Aragón (IIS Aragón)

^5^ CIBERobn (Centro de Investigación Biomédica en Red Fisiopatología de la Obesidad y Nutrición), Institute of Health Carlos III, Madrid, Spain

^6^ Hydration Science Lab, University of Arkansas, Fayetteville, AR, USA

^7^ Division of Endocrinology, University of Arkansas for Medical Sciences, Little Rock, AR, USA

^8^ Hospital Infantil de México Federico Gómez, México City, México

^9^ Nutrition International, Ottawa, Canada

^10^ Human Nutrition Unit, Hospital Universitari de Sant Joan de Reus, Faculty of Medicine and Health Sciences, Institut d’Investigació Sanitària Pere Virgili, Biochemistry and Biotechnology Department, Universitat Rovira i Virgili Reus, Spain

*** Corresponding author:** Isabelle GUELINCKX, Hydration and Health department, Danone Nutricia Research, Route Départemental 128, 91767 Palaiseau, France; isabelle.guelinckx@danone.com

**ONLINE RESOURCE**

**Table S1** General characteristics of the survey population, by country and age group

| Age group | Country | Gender |  | Age (years) | BMI (kg/m²) |
| --- | --- | --- | --- | --- | --- |
|  |  | **Male** | **Female** |  |  |
| **4-9 years** |  |  |  |  |  |
|  | Mexico (n=293) | 140 (48) | 153 (52) | 6.5 ± 1.7 | 25.7 ± 15.1 |
|  | Brazil (n=146) | 68 (47) | 78 (53) | 6.6 ± 1.8 | 18.7 ± 4.3 |
|  | Argentina (n=173) | 114 (66) | 59 (34) | 6.4 ± 1.7 | 19.3 ± 6.2 |
|  | Uruguay (n=121) | 57 (47) | 64 (53) | 7.7 ± 0.9 | 18.9 ± 4.5 |
|  | China (n=279) | 143 (51) | 136 (49) | 6.3 ± 1.6 | 19.4 ± 6.3 |
|  | Indonesia (n=388) | 244 (63) | 144 (37) | 6.4 ± 1.7 | 21.7 ± 14.4 |
| **10-17 years** |  |  |  |  |  |
|  | Mexico (n=376) | 212 (56) | 164 (44) | 13.5 ± 2.4 | 22.8 ± 5.8 |
|  | Brazil (n=194) | 62 (32) | 132 (68) | 13.6 ± 2.3 | 21.4 ± 5.2 |
|  | Argentina (n=219) | 130 (59) | 89 (41) | 13.5 ± 2.2 | 21.3 ± 3.8 |
|  | Uruguay (n=144) | 74 (51) | 70 (49) | 12.3 ± 2.3 | 20.7 ± 8.5 |
|  | China (n=370) | 187 (51) | 183 (49) | 13.7 ± 2.4 | 21.2 ± 5.9 |
|  | Indonesia (n=478) | 278 (58) | 200 (42) | 13.4 ± 2.3 | 22.3 ± 16.2 |
| **≥ 18 years** |  |  |  |  |  |
|  | Mexico (n=1677) | 746 (44) | 931 (56) | 37.6 ± 13.1 | 26.7 ± 4.9 |
|  | Brazil (n=477) | 224 (47) | 253 (53) | 39.5 ± 14.0 | 27.0 ± 6.8 |
|  | Argentina (n=1089) | 464 (43) | 625 (57) | 38.5 ± 13.3 | 26.0 ± 5.6 |
|  | Uruguay (n=554) | 278 (50) | 276 (50) | 37.7 ± 13.3 | 25.7 ± 10.3 |
|  | China (n=1584) | 790 (50) | 794 (50) | 33.7 ± 10.2 | 23.2 ± 5.6 |
|  | Indonesia (n=2778) | 1256 (45) | 1522 (55) | 35.5 ± 12.1 | 23.2 ± 5.5 |

*BMI* body mass index

Age and BMI presented as mean ± standard deviation and gender as number and proportion(%)

**Table S2a** Mean (SEM) intake of different fluid types (mL/day) according to meal occasions among children (4-9 years), by country

| Country | Occasions | Water | | Milk & derivatives | | Hot beverages | | SSB | | 100% FJ | | A/NSB | | Alcoholic beverages | | Other beverages | | TFI | |
| --- | --- | --- | --- | --- | --- | --- | --- | --- | --- | --- | --- | --- | --- | --- | --- | --- | --- | --- | --- |
| **Mexico (n=293)** | Daily total | 384 | ±26 | 315 | ±16 | 37 | ±6 | 458 | ±23 | 19 | ±3 | 11 | ±3 | 0 | ±0 | 7 | ±3 | 1232 | ±39 |
|  | Meals | 115 | ±11 | 177 | ±13 | 19 | ±4 | 291 | ±18 | 11 | ±2 | 9 | ±3 | 0 | ±0 | 3 | ±2 | 625 | ±25 |
|  | Outside of meals | 68 | ±12 | 51 | ±6 | 6 | ±2 | 69 | ±7 | 3 | ±1 | 1 | ±0 | 0 | ±0 | 0 | ±0 | 198 | ±17 |
|  | Without food | 202 | ±19 | 87 | ±9 | 12 | ±3 | 98 | ±11 | 6 | ±2 | 1 | ±1 | 0 | ±0 | 3 | ±1 | 409 | ±27 |
| **Brazil (n=146)** | Daily total | 455 | ±26 | 336 | ±21 | 44 | ±6 | 434 | ±25 | 120 | ±22 | 9 | ±3 | 0 | ±0 | 15 | ±4 | 1414 | ±56 |
|  | Meals | 21 | ±4 | 63 | ±8 | 16 | ±4 | 232 | ±17 | 49 | ±6 | 6 | ±2 | 0 | ±0 | 5 | ±2 | 393 | ±24 |
|  | Outside of meals | 19 | ±7 | 71 | ±9 | 12 | ±3 | 79 | ±9 | 40 | ±19 | 2 | ±1 | 0 | ±0 | 3 | ±1 | 227 | ±27 |
|  | Without food | 386 | ±24 | 183 | ±16 | 13 | ±2 | 104 | ±12 | 27 | ±5 | 2 | ±1 | 0 | ±0 | 6 | ±2 | 722 | ±33 |
|  | Unspecified | 28 | ±4 | 19 | ±3 | 3 | ±1 | 18 | ±4 | 4 | ±1 | 0 | ±0 | 0 | ±0 | 0 | ±0 | 72 | ±9 |
| **Argentina (n=173)** | Daily total | 435 | ±41 | 364 | ±20 | 141 | ±14 | 711 | ±46 | 19 | ±5 | 133 | ±20 | 1 | ±0 | 2 | ±1 | 1807 | ±61 |
|  | Meals | 174 | ±23 | 106 | ±15 | 34 | ±6 | 462 | ±31 | 9 | ±4 | 94 | ±16 | 1 | ±0 | 1 | ±1 | 881 | ±37 |
|  | Outside of meals | 46 | ±8 | 175 | ±15 | 89 | ±12 | 122 | ±13 | 5 | ±2 | 25 | ±5 | 0 | ±0 | 0 | ±0 | 463 | ±25 |
|  | Without food | 215 | ±24 | 83 | ±11 | 17 | ±4 | 127 | ±16 | 6 | ±2 | 14 | ±3 | 0 | ±0 | 0 | ±0 | 463 | ±31 |
| **Uruguay (n=121)** | Daily total | 535 | ±34 | 418 | ±28 | 1 | ±1 | 687 | ±44 | 4 | ±4 | 8 | ±5 | 0 | ±0 | 40 | ±13 | 1693 | ±52 |
|  | Meals | 242 | ±24 | 8 | ±3 | 0 | ±0 | 449 | ±35 | 0 | ±0 | 1 | ±1 | 0 | ±0 | 0 | ±0 | 701 | ±32 |
|  | Outside of meals | 66 | ±16 | 386 | ±29 | 0 | ±0 | 113 | ±20 | 4 | ±4 | 6 | ±4 | 0 | ±0 | 33 | ±11 | 608 | ±34 |
|  | Without food | 227 | ±25 | 24 | ±6 | 1 | ±1 | 125 | ±24 | 0 | ±0 | 1 | ±1 | 0 | ±0 | 6 | ±3 | 384 | ±35 |
| **China (n=279)** | Daily total | 494 | ±21 | 269 | ±12 | 11 | ±2 | 128 | ±12 | 61 | ±7 | 1 | ±0 | 0 | ±0 | 2 | ±1 | 966 | 30 |
|  | Meals | 89 | ±8 | 106 | ±6 | 3 | ±1 | 45 | ±5 | 22 | ±3 | 0 | ±0 | 0 | ±0 | 1 | ±1 | 266 | ±13 |
|  | Outside of meals | 32 | ±5 | 22 | ±3 | 2 | ±1 | 18 | ±4 | 9 | ±2 | 1 | ±0 | 0 | ±0 | 0 | ±0 | 83 | ±11 |
|  | Without food | 373 | ±17 | 141 | ±9 | 6 | ±2 | 65 | ±6 | 31 | ±4 | 0 | ±0 | 0 | ±0 | 1 | ±0 | 617 | ±22 |
| **Indonesia (n=388)** | Daily total | 1663 | ±42 | 254 | ±16 | 68 | ±9 | 167 | ±12 | 6 | ±2 | 2 | ±1 | 0 | ±0 | 5 | ±2 | 2165 | ±45 |
|  | Meals | 439 | ±17 | 38 | ±5 | 18 | ±3 | 24 | ±3 | 1 | ±0 | 0 | ±0 | 0 | ±0 | 0 | ±0 | 520 | ±18 |
|  | Outside of meals | 263 | ±20 | 49 | ±5 | 21 | ±4 | 39 | ±5 | 1 | ±0 | 0 | ±0 | 0 | ±0 | 1 | ±0 | 373 | ±23 |
|  | Without food | 961 | ±36 | 168 | ±14 | 28 | ±4 | 105 | ±9 | 5 | ±1 | 2 | ±1 | 0 | ±0 | 4 | ±2 | 1272 | ±40 |

*SEM* Standard error of the mean, *SSB* sugar sweetened beverages, *FJ* fruit juices, *A/NSB* artificial/non-nutritive sweeteners beverages, *TFI* total fluid intake

**Table S2b** Mean (SEM) intake of different fluid types (mL/day) according to meal occasions among adolescents (10-17 years), by country

| **Country** | Occasions | Water | | Milk & derivatives | | Hot beverages | | SSB | | 100% FJ | | A/NSB | | Alcoholic beverages | | Other beverages | | TFI | |
| --- | --- | --- | --- | --- | --- | --- | --- | --- | --- | --- | --- | --- | --- | --- | --- | --- | --- | --- | --- |
| **Mexico (n=376)** | Daily total | 639 | ±35 | 211 | ±12 | 102 | ±10 | 667 | ±29 | 25 | ±4 | 15 | ±3 | 4 | ±2 | 15 | ±4 | 1679 | ±47 |
|  | Meals | 197 | ±17 | 107 | ±9 | 56 | ±7 | 422 | ±23 | 14 | ±3 | 8 | ±2 | 2 | ±1 | 4 | ±2 | 809 | ±31 |
|  | Outside of meals | 98 | ±12 | 48 | ±6 | 19 | ±4 | 113 | ±13 | 4 | ±2 | 2 | ±1 | 0 | ±0 | 5 | ±2 | 289 | ±19 |
|  | Without food | 343 | ±26 | 56 | ±6 | 27 | ±4 | 133 | ±11 | 8 | ±2 | 6 | ±2 | 2 | ±1 | 5 | ±2 | 581 | ±32 |
| **Brazil (n=194)** | Daily total | 630 | ±41 | 207 | ±17 | 102 | ±11 | 600 | ±32 | 121 | ±17 | 28 | ±5 | 21 | ±7 | 5 | ±2 | 1713 | ±69 |
|  | Meals | 48 | ±7 | 55 | ±6 | 37 | ±6 | 288 | ±20 | 47 | ±7 | 12 | ±3 | 0 | ±0 | 0 | ±0 | 487 | ±29 |
|  | Outside of meals | 35 | ±7 | 51 | ±8 | 25 | ±4 | 110 | ±10 | 23 | ±4 | 5 | ±1 | 3 | ±2 | 2 | ±1 | 253 | ±22 |
|  | Without food | 508 | ±31 | 88 | ±9 | 37 | ±8 | 181 | ±16 | 44 | ±9 | 10 | ±2 | 17 | ±6 | 2 | ±1 | 888 | ±41 |
|  | Unspecified | 39 | ±10 | 13 | ±4 | 4 | ±1 | 21 | ±5 | 7 | ±2 | 1 | ±0 | 0 | ±0 | 0 | ±0 | 85 | ±16 |
| **Argentina (n=219)** | Daily total | 454 | ±31 | 222 | ±17 | 239 | ±19 | 818 | ±42 | 17 | ±5 | 128 | ±19 | 17 | ±5 | 2 | ±1 | 1897 | ±51 |
|  | Meals | 202 | ±21 | 47 | ±7 | 42 | ±6 | 561 | ±32 | 8 | ±4 | 97 | ±15 | 6 | ±3 | 0 | ±0 | 965 | ±33 |
|  | Outside of meals | 57 | ±9 | 130 | ±15 | 137 | ±13 | 111 | ±11 | 3 | ±1 | 13 | ±4 | 2 | ±2 | 0 | ±0 | 454 | ±26 |
|  | Without food | 195 | ±17 | 45 | ±7 | 60 | ±10 | 145 | ±16 | 6 | ±2 | 18 | ±4 | 8 | ±4 | 2 | ±1 | 479 | ±29 |
| **Uruguay (n=144)** | Daily total | 561 | ±51 | 318 | ±26 | 164 | ±31 | 521 | ±41 | 21 | ±9 | 57 | ±14 | 2 | ±1 | 25 | ±6 | 1668 | ±68 |
|  | Meals | 240 | ±33 | 18 | ±6 | 24 | ±9 | 323 | ±30 | 14 | ±7 | 32 | ±9 | 1 | ±1 | 11 | ±5 | 662 | ±39 |
|  | Outside of meals | 65 | ±12 | 259 | ±25 | 63 | ±15 | 100 | ±19 | 7 | ±5 | 6 | ±3 | 0 | ±0 | 13 | ±4 | 514 | ±31 |
|  | Without food | 256 | ±37 | 41 | ±11 | 76 | ±20 | 98 | ±16 | 0 | ±0 | 19 | ±7 | 1 | ±1 | 1 | ±1 | 492 | ±48 |
| **China (n=370)** | Daily total | 632 | ±27 | 189 | ±8 | 37 | ±6 | 253 | ±13 | 45 | ±4 | 7 | ±1 | 13 | ±4 | 1 | ±1 | 1177 | ±31 |
|  | Meals | 124 | ±8 | 99 | ±5 | 10 | ±2 | 78 | ±6 | 19 | ±2 | 2 | ±1 | 7 | ±2 | 1 | ±1 | 341 | ±13 |
|  | Outside of meals | 35 | ±7 | 11 | ±2 | 3 | ±1 | 20 | ±3 | 5 | ±1 | 0 | ±0 | 3 | ±2 | 0 | ±0 | 77 | ±9 |
|  | Without food | 474 | ±22 | 80 | ±5 | 24 | ±5 | 155 | ±9 | 21 | ±2 | 4 | ±1 | 3 | ±1 | 0 | ±0 | 759 | ±26 |
| **Indonesia (n=478)** | Daily total | 1933 | ±43 | 136 | ±11 | 121 | ±9 | 282 | ±16 | 9 | ±2 | 3 | ±2 | 0 | ±0 | 4 | ±1 | 2488 | ±49 |
|  | Meals | 527 | ±19 | 23 | ±4 | 34 | ±4 | 51 | ±5 | 1 | ±1 | 0 | ±0 | 0 | ±0 | 1 | ±0 | 637 | ±21 |
|  | Outside of meals | 270 | ±18 | 40 | ±6 | 35 | ±4 | 77 | ±7 | 2 | ±1 | 1 | ±1 | 0 | ±0 | 1 | ±0 | 426 | ±23 |
|  | Without food | 1136 | ±36 | 73 | ±7 | 52 | ±5 | 155 | ±11 | 5 | ±2 | 2 | ±1 | 0 | ±0 | 3 | ±1 | 1425 | ±40 |

*SEM* Standard error of the mean, *SSB* sugar sweetened beverages, *FJ* fruit juices, *A/NSB* artificial/non-nutritive sweeteners beverages, *TFI* total fluid intake

**Table S2c** Mean (SEM) intake of different fluid types (mL/day) according to meal occasions among adults (≥18 years), by country

| Country | Occasions | Water | | Milk & derivatives | | Hot beverages | | SSB | | 100% FJ | | A/NSB | | Alcoholic beverages | | | Other beverages | | | TFI | | |
| --- | --- | --- | --- | --- | --- | --- | --- | --- | --- | --- | --- | --- | --- | --- | --- | --- | --- | --- | --- | --- | --- | --- |
| Mexico (n=1677) | Daily total | 673 | ±18 | 132 | ±5 | 210 | ±7 | 655 | ±13 | 35 | ±3 | 23 | ±2 | 22 | ±2 | 6 | | ±1 | 1754 | | ±24 |  |
|  | Meals | 192 | ±9 | 61 | ±3 | 111 | ±5 | 438 | ±10 | 19 | ±2 | 13 | ±2 | 6 | ±1 | 2 | | ±0 | 842 | | ±14 |  |
|  | Outside of meals | 63 | ±4 | 25 | ±2 | 42 | ±3 | 80 | ±4 | 4 | ±1 | 3 | ±1 | 7 | ±1 | 1 | | ±0 | 225 | | ±8 |  |
|  | Without food | 418 | ±14 | 46 | ±3 | 57 | ±4 | 137 | ±6 | 13 | ±2 | 7 | ±1 | 8 | ±1 | 3 | | ±0 | 688 | | ±17 |  |
| Brazil (n=477) | Daily total | 756 | ±28 | 92 | ±7 | 199 | ±10 | 490 | ±19 | 110 | ±7 | 23 | ±4 | 142 | ±12 | 10 | | ±2 | 1822 | | ±46 |  |
|  | Meals | 60 | ±7 | 25 | ±3 | 64 | ±5 | 258 | ±12 | 53 | ±5 | 11 | ±2 | 22 | ±4 | 1 | | ±0 | 493 | | ±18 |  |
|  | Outside of meals | 28 | ±4 | 17 | ±2 | 42 | ±3 | 74 | ±6 | 17 | ±2 | 6 | ±2 | 28 | ±4 | 0 | | ±0 | 211 | | ±12 |  |
|  | Without food | 632 | ±23 | 47 | ±4 | 87 | ±7 | 143 | ±9 | 38 | ±3 | 6 | ±1 | 88 | ±8 | 8 | | ±2 | 1049 | | ±33 |  |
|  | Unspecified | 36 | ±6 | 3 | ±1 | 6 | ±1 | 15 | ±2 | 2 | ±0 | 1 | ±0 | 4 | ±1 | 1 | | ±0 | 68 | | ±8 |  |
| Argentina (n=1089) | Daily total | 581 | ±20 | 60 | ±4 | 827 | ±17 | 488 | ±16 | 22 | ±3 | 160 | ±10 | 128 | ±8 | 6 | | ±1 | 2272 | | ±31 |  |
|  | Unspecified | 36 | ±6 | 3 | ±1 | 6 | ±1 | 15 | ±2 | 2 | ±0 | 1 | ±0 | 4 | ±1 | 1 | | ±0 | 68 | | ±8 |  |
|  | Meals | 244 | ±11 | 16 | ±2 | 143 | ±8 | 367 | ±12 | 8 | ±2 | 128 | ±8 | 79 | ±5 | 3 | | ±1 | 987 | | ±17 |  |
|  | Outside of meals | 36 | ±4 | 21 | ±2 | 378 | ±12 | 42 | ±3 | 5 | ±1 | 10 | ±2 | 15 | ±2 | 0 | | ±0 | 506 | | ±13 |  |
|  | Without food | 301 | ±13 | 24 | ±3 | 307 | ±13 | 79 | ±6 | 9 | ±2 | 22 | ±3 | 34 | ±4 | 3 | | ±1 | 779 | | ±21 |  |
| Uruguay (n=554) | Daily total | 493 | ±22 | 83 | ±6 | 873 | ±35 | 359 | ±17 | 26 | ±4 | 103 | ±10 | 48 | ±6 | 14 | | ±3 | 1999 | | ±43 |  |
|  | Meals | 208 | ±14 | 11 | ±2 | 78 | ±10 | 220 | ±12 | 7 | ±2 | 76 | ±8 | 30 | ±5 | 8 | | ±2 | 639 | | ±20 |  |
|  | Outside of meals | 43 | ±6 | 52 | ±5 | 418 | ±27 | 48 | ±6 | 9 | ±2 | 10 | ±3 | 8 | ±1 | 2 | | ±1 | 590 | | ±29 |  |
|  | Without food | 241 | ±15 | 20 | ±3 | 376 | ±24 | 91 | ±9 | 10 | ±2 | 17 | ±3 | 10 | ±2 | 4 | | ±2 | 769 | | ±30 |  |
| China (n=1584) | Daily total | 751 | ±16 | 132 | ±3 | 136 | ±7 | 261 | ±7 | 35 | ±2 | 7 | ±1 | 62 | ±4 | 2 | | ±0 | 1387 | | ±18 |  |
|  | Meals | 147 | ±6 | 73 | ±2 | 21 | ±1 | 87 | ±3 | 17 | ±1 | 3 | ±1 | 47 | ±3 | 1 | | ±0 | 395 | | ±8 |  |
|  | Outside of meals | 31 | ±3 | 6 | ±1 | 8 | ±1 | 18 | ±1 | 2 | ±0 | 0 | ±0 | 4 | ±1 | 0 | | ±0 | 70 | | ±4 |  |
|  | Without food | 573 | ±13 | 54 | ±2 | 107 | ±6 | 156 | ±5 | 16 | ±1 | 4 | ±1 | 11 | ±1 | 1 | | ±0 | 922 | | ±15 |  |
| **Indonesia (n=2778)** | Daily total | 2164 | ±20 | 52 | ±3 | 263 | ±6 | 227 | ±7 | 12 | ±1 | 2 | ±0 | 0 | ±0 | 2 | | ±0 | 2721 | | ±22 |  |
|  | Meals | 598 | ±9 | 8 | ±1 | 57 | ±3 | 49 | ±2 | 2 | ±0 | 0 | ±0 | 0 | ±0 | 0 | | ±0 | 715 | | ±10 |  |
|  | Outside of meals | 273 | ±8 | 13 | ±1 | 85 | ±3 | 58 | ±3 | 3 | ±0 | 1 | ±0 | 0 | ±0 | 0 | | ±0 | 433 | | ±10 |  |
|  | Without food | 1292 | ±16 | 31 | ±2 | 121 | ±4 | 120 | ±4 | 7 | ±1 | 1 | ±0 | 0 | ±0 | 1 | | ±0 | 1573 | | ±18 |  |

*SEM* Standard error of the mean, *SSB* sugar sweetened beverages, *FJ* fruit juices, *A/NSB* artificial/non-nutritive sweeteners beverages, *TFI* total fluid intake

**Table S3a** Median (P25-P75) intake of different fluid types (mL/day) according to drinking occasions among children (4-9 years), by country

| **Occasions** | **Water** | **Milk & derivatives** | **Hot beverages** | **SSB** | **100% FJ** | **A/NSB** | **Alcoholic beverages** | **Other beverages** | **TFI** |
| --- | --- | --- | --- | --- | --- | --- | --- | --- | --- |
| **Mexico (n=293)** |  |  |  |  |  |  |  |  |  |
| Daily total | 252 (90-505) | 265 (111-451) | 0 (0-28) | 391 (185-607) | 0 (0-0) | 0 (0-0) | 0 (0-0) | 0 (0-0) | 1061 (751-1490) |
| Meals | 43 (0-143) | 103 (0-268) | 0 (0-0) | 214 (72-387) | 0 (0-0) | 0 (0-0) | 0 (0-0) | 0 (0-0) | 550 (293-878) |
| Outside of meals | 0 (0-60) | 0 (0-57) | 0 (0-0) | 0 (0-86) | 0 (0-0) | 0 (0-0) | 0 (0-0) | 0 (0-0) | 86 (0-272) |
| Without food | 94 (0-271) | 9 (0-107) | 0 (0-0) | 36 (0-132) | 0 (0-0) | 0 (0-0) | 0 (0-0) | 0 (0-0) | 284 (93-533) |
| **Brazil ^a^ (n=146)** |  |  |  |  |  |  |  |  |  |
| Daily total | 369 (238-609) | 302 (152-468) | 0 (0-63) | 396 (197-603) | 55 (0-158) | 0 (0-0) | 0 (0-0) | 0 (0-0) | 1297 (957-1632) |
| Meals | 0 (0-27) | 27 (0-89) | 0 (0-0) | 211 (65-349) | 9 (0-72) | 0 (0-0) | 0 (0-0) | 0 (0-0) | 330 (170-551) |
| Outside of meals | 0 (0-2) | 29 (0-107) | 0 (0-0) | 34 (0-121) | 0 (0-29) | 0 (0-0) | 0 (0-0) | 0 (0-0) | 153 (52-299) |
| Without food | 317 (178-536) | 138 (36-268) | 0 (0-0) | 63 (7-141) | 0 (0-29) | 0 (0-0) | 0 (0-0) | 0 (0-0) | 619 (462-869) |
| **Argentina (n=173)** |  |  |  |  |  |  |  |  |  |
| Daily total | 271 (79-607) | 343 (154-517) | 54 (0-211) | 545 (254-1062) | 0 (0-0) | 0 (0-156) | 0 (0-0) | 0 (0-0) | 1633 (1185-2344) |
| Meals | 43 (0-214) | 0 (0-115) | 0 (0-0) | 380 (133-672) | 0 (0-0) | 0 (0-86) | 0 (0-0) | 0 (0-0) | 800 (493-1193) |
| Outside of meals | 0 (0-51) | 114 (0-293) | 0 (0-119) | 50 (0-187) | 0 (0-0) | 0 (0-0) | 0 (0-0) | 0 (0-0) | 429 (206-685) |
| Without food | 103 (0-286) | 0 (0-103) | 0 (0-0) | 51 (0-166) | 0 (0-0) | 0 (0-0) | 0 (0-0) | 0 (0-0) | 343 (190-658) |
| **Uruguay (n=121)** |  |  |  |  |  |  |  |  |  |
| Daily total | 500 (250-768) | 429 (232-500) | 0 (0-0) | 570 (275-1026) | 0 (0-0) | 0 (0-0) | 0 (0-0) | 0 (0-0) | 1639 (1311-1904) |
| Meals | 163 (0-456) | 0 (0-0) | 0 (0-0) | 350 (200-666) | 0 (0-0) | 0 (0-0) | 0 (0-0) | 0 (0-0) | 686 (517-846) |
| Outside of meals | 0 (0-54) | 357 (171-500) | 0 (0-0) | 0 (0-143) | 0 (0-0) | 0 (0-0) | 0 (0-0) | 0 (0-0) | 523 (377-730) |
| Without food | 150 (0-338) | 0 (0-0) | 0 (0-0) | 0 (0-150) | 0 (0-0) | 0 (0-0) | 0 (0-0) | 0 (0-0) | 316 (100-591) |
| **China (n=279)** |  |  |  |  |  |  |  |  |  |
| Daily total | 403 (245-658) | 227 (136-356) | 0 (0-0) | 71 (0-179) | 21 (0-81) | 0 (0-0) | 0 (0-0) | 0 (0-0) | 839 (611-1196) |
| Meals | 43 (0-107) | 84 (31-157) | 0 (0-0) | 9 (0-68) | 0 (0-28) | 0 (0-0) | 0 (0-0) | 0 (0-0) | 216 (133-341) |
| Outside of meals | 0 (0-34) | 0 (0-26) | 0 (0-0) | 0 (0-0) | 0 (0-0) | 0 (0-0) | 0 (0-0) | 0 (0-0) | 16 (0-71) |
| Without food | 291 (156-501) | 107 (36-200) | 0 (0-0) | 33 (0-99) | 0 (0-43) | 0 (0-0) | 0 (0-0) | 0 (0-0) | 541 (363-819) |
| **Indonesia (n=388)** |  |  |  |  |  |  |  |  |  |
| Daily total | 1449 (1037-2298) | 150 (0-377) | 0 (0-64) | 69 (0-240) | 0 (0-0) | 0 (0-0) | ND | 0 (0-0) | 2074 (1431-2880) |
| Meals | 429 (208-617) | 0 (0-26) | 0 (0-0) | 0 (0-0) | 0 (0-0) | 0 (0-0) | ND | 0 (0-0) | 498 (277-716) |
| Outside of meals | 129 (0-353) | 0 (0-41) | 0 (0-0) | 0 (0-34) | 0 (0-0) | 0 (0-0) | ND | 0 (0-0) | 255 (63-512) |
| Without food | 780 (363-1435) | 57 (0-240) | 0 (0-0) | 19 (0-143) | 0 (0-0) | 0 (0-0) | ND | 0 (0-0) | 1135 (625-1839) |

*SSB* sugared sweetened beverages, *FJ* fruit juices, *A/NSB* artificial/non-nutritive sweeteners beverages, *TFI* total fluid intake

Data presented as median (25, 75^th^ percentiles)

**Table S3b** Median (P25-P75) intake of different fluid types (mL/day) according to drinking occasions among adolescents (10-17 years**)**, by country

| **Occasions** | **Water** | **Milk & derivatives** | **Hot beverages** | **SSB** | **100% FJ** | **A/NSB** | **Alcoholic beverages** | **Other beverages** | **TFI** |
| --- | --- | --- | --- | --- | --- | --- | --- | --- | --- |
| **Mexico (n=376)** |  |  |  |  |  |  |  |  |  |
| Daily total | 391 (180-963) | 154 (9-320) | 0 (0-131) | 524 (258-908) | 0 (0-0) | 0 (0-0) | 0 (0-0) | 0 (0-0) | 1462 (1011-2121) |
| Meals | 69 (0-245) | 21 (0-137) | 0 (0-43) | 323 (129-575) | 0 (0-0) | 0 (0-0) | 0 (0-0) | 0 (0-0) | 695 (391-1047) |
| Outside of meals | 0 (0-96) | 0 (0-43) | 0 (0-0) | 0 (0-125) | 0 (0-0) | 0 (0-0) | 0 (0-0) | 0 (0-0) | 159 (0-419) |
| Without food | 154 (0-443) | 0 (0-68) | 0 (0-0) | 43 (0-172) | 0 (0-0) | 0 (0-0) | 0 (0-0) | 0 (0-0) | 430 (141-771) |
| **Brazil ^a^ (n=194)** |  |  |  |  |  |  |  |  |  |
| Daily total | 505 (278-778) | 156 (36-265) | 31 (0-149) | 499 (262-811) | 42 (0-157) | 0 (0-36) | 0 (0-0) | 0 (0-0) | 1491 (1030-2231) |
| Meals | 0 (0-54) | 8 (0-79) | 0 (0-36) | 205 (71-440) | 0 (0-54) | 0 (0-0) | 0 (0-0) | 0 (0-0) | 403 (189-679) |
| Outside of meals | 0 (0-20) | 0 (0-57) | 0 (0-25) | 52 (0-163) | 0 (0-20) | 0 (0-0) | 0 (0-0) | 0 (0-0) | 157 (63-349) |
| Without food | 405 (221-650) | 41 (0-125) | 0 (0-36) | 108 (35-252) | 0 (0-37) | 0 (0-0) | 0 (0-0) | 0 (0-0) | 781 (423-1192) |
| **Argentina (n=219)** |  |  |  |  |  |  |  |  |  |
| Daily total | 346 (93-675) | 144 (0-361) | 179 (0-357) | 686 (388-1147) | 0 (0-0) | 0 (0-100) | 0 (0-0) | 0 (0-0) | 1780 (1362-2400) |
| Meals | 34 (0-300) | 0 (0-26) | 0 (0-36) | 480 (189-791) | 0 (0-0) | 0 (0-54) | 0 (0-0) | 0 (0-0) | 889 (592-1320) |
| Outside of meals | 0 (0-50) | 34 (0-187) | 56 (0-227) | 43 (0-164) | 0 (0-0) | 0 (0-0) | 0 (0-0) | 0 (0-0) | 393 (146-679) |
| Without food | 100 (0-310) | 0 (0-43) | 0 (0-45) | 63 (0-204) | 0 (0-0) | 0 (0-0) | 0 (0-0) | 0 (0-0) | 392 (191-673) |
| **Uruguay (n=144)** |  |  |  |  |  |  |  |  |  |
| Daily total | 434 (155-798) | 240 (63-498) | 0 (0-87) | 390 (194-749) | 0 (0-0) | 0 (0-0) | 0 (0-0) | 0 (0-0) | 1544 (1088-2115) |
| Meals | 79 (0-428) | 0 (0-0) | 0 (0-0) | 209 (73-489) | 0 (0-0) | 0 (0-0) | 0 (0-0) | 0 (0-0) | 650 (350-829) |
| Outside of meals | 0 (0-53) | 165 (0-393) | 0 (0-0) | 0 (0-137) | 0 (0-0) | 0 (0-0) | 0 (0-0) | 0 (0-0) | 454 (208-747) |
| Without food | 92 (0-367) | 0 (0-0) | 0 (0-0) | 0 (0-128) | 0 (0-0) | 0 (0-0) | 0 (0-0) | 0 (0-0) | 290 (109-734) |
| **China (n=370)** |  |  |  |  |  |  |  |  |  |
| Daily total | 518 (303-777) | 161 (68-278) | 0 (0-36) | 205 (64-363) | 0 (0-71) | 0 (0-0) | 0 (0-0) | 0 (0-0) | 1071 (775-1408) |
| Meals | 71 (12-175) | 75 (21-157) | 0 (0-0) | 38 (0-112) | 0 (0-15) | 0 (0-0) | 0 (0-0) | 0 (0-0) | 300 (194-428) |
| Outside of meals | 0 (0-22) | 0 (0-0) | 0 (0-0) | 0 (0-0) | 0 (0-0) | 0 (0-0) | 0 (0-0) | 0 (0-0) | 0 (0-79) |
| Without food | 377 (214-589) | 37 (0-126) | 0 (0-20) | 107 (16-229) | 0 (0-25) | 0 (0-0) | 0 (0-0) | 0 (0-0) | 656 (437-982) |
| **Indonesia (n=478)** |  |  |  |  |  |  |  |  |  |
| Daily total | 1856 (1222-2465) | 4 (0-214) | 31 (0-192) | 173 (0-412) | 0 (0-0) | 0 (0-0) | ND | 0 (0-0) | 2422 (1651-3138) |
| Meals | 480 (240-720) | 0 (0-0) | 0 (0-27) | 0 (0-43) | 0 (0-0) | 0 (0-0) | ND | 0 (0-0) | 596 (337-846) |
| Outside of meals | 154 (0-346) | 0 (0-0) | 0 (0-26) | 0 (0-88) | 0 (0-0) | 0 (0-0) | ND | 0 (0-0) | 274 (83-599) |
| Without food | 999 (514-1633) | 0 (0-78) | 0 (0-50) | 57 (0-217) | 0 (0-0) | 0 (0-0) | ND | 0 (0-0) | 1344 (736-2028) |

*SSB* sugared sweetened beverages, *FJ* fruit juices, *A/NSB* artificial/non-nutritive sweeteners beverages, *TFI* total fluid intake

Data presented as median (25, 75^th^ percentiles)**Table S3c** Median (P25-P75) intake of different fluid types (mL/day) according to drinking occasions among adults (≥ 18 years), by country

| **Occasions** | **Water** | **Milk & derivatives** | **Hot beverages** | **SSB** | **100% FJ** | **A/NSB** | **Alcoholic beverages** | **Other beverages** | **TFI** |
| --- | --- | --- | --- | --- | --- | --- | --- | --- | --- |
| **Mexico (n=1677)** |  |  |  |  |  |  |  |  |  |
| Daily total | 450 (180-900) | 60 (0-186) | 121 (0-286) | 531 (300-895) | 0 (0-0) | 0 (0-0) | 0 (0-0) | 0 (0-0) | 1496 (1060-2150) |
| Meals | 57 (0-235) | 0 (0-69) | 18 (0-143) | 356 (137-605) | 0 (0-0) | 0 (0-0) | 0 (0-0) | 0 (0-0) | 739 (420-1142) |
| Outside of meals | 0 (0-43) | 0 (0-0) | 0 (0-18) | 0 (0-86) | 0 (0-0) | 0 (0-0) | 0 (0-0) | 0 (0-0) | 89 (0-320) |
| Without food | 214 (21-593) | 0 (0-43) | 0 (0-46) | 43 (0-186) | 0 (0-0) | 0 (0-0) | 0 (0-0) | 0 (0-0) | 480 (193-970) |
| **Brazil (n=477)** |  |  |  |  |  |  |  |  |  |
| Daily total | 599 (349-1004) | 34 (0-117) | 150 (51-263) | 409 (195-669) | 50 (0-165) | 0 (0-0) | 0 (0-202) | 0 (0-0) | 1568 (1098-2333) |
| Meals | 0 (0-64) | 0 (0-18) | 14 (0-86) | 178 (64-386) | 0 (0-70) | 0 (0-0) | 0 (0-0) | 0 (0-0) | 411 (213-689) |
| Outside of meals | 0 (0-21) | 0 (0-0) | 0 (0-54) | 18 (0-91) | 0 (0-0) | 0 (0-0) | 0 (0-0) | 0 (0-0) | 130 (40-291) |
| Without food | 489 (271-838) | 0 (0-54) | 36 (0-121) | 80 (0-197) | 0 (0-43) | 0 (0-0) | 0 (0-109) | 0 (0-0) | 873 (574-1303) |
| **Argentina (n=1089)** |  |  |  |  |  |  |  |  |  |
| Daily total | 386 (96-855) | 0 (0-46) | 732 (431-1136) | 338 (82-754) | 0 (0-0) | 0 (0-154) | 0 (0-150) | 0 (0-0) | 2133 (1524-2865) |
| Meals | 64 (0-377) | 0 (0-0) | 0 (0-214) | 236 (39-570) | 0 (0-0) | 0 (0-125) | 0 (0-100) | 0 (0-0) | 896 (558-1330) |
| Outside of meals | 0 (0-18) | 0 (0-0) | 286 (64-557) | 0 (0-25) | 0 (0-0) | 0 (0-0) | 0 (0-0) | 0 (0-0) | 437 (157-745) |
| Without food | 150 (0-417) | 0 (0-0) | 159 (0-439) | 0 (0-80) | 0 (0-0) | 0 (0-0) | 0 (0-0) | 0 (0-0) | 607 (282-1081) |
| **Uruguay (n=554)** |  |  |  |  |  |  |  |  |  |
| Daily total | 325 (129-721) | 0 (0-125) | 699 (184-1356) | 215 (30-559) | 0 (0-0) | 0 (0-110) | 0 (0-0) | 0 (0-0) | 1873 (1194-2593) |
| Meals | 34 (0-294) | 0 (0-0) | 0 (0-15) | 104 (0-343) | 0 (0-0) | 0 (0-50) | 0 (0-0) | 0 (0-0) | 506 (286-877) |
| Outside of meals | 0 (0-0) | 0 (0-12) | 103 (0-615) | 0 (0-14) | 0 (0-0) | 0 (0-0) | 0 (0-0) | 0 (0-0) | 356 (70-885) |
| Without food | 103 (0-335) | 0 (0-0) | 86 (0-600) | 0 (0-81) | 0 (0-0) | 0 (0-0) | 0 (0-0) | 0 (0-0) | 563 (236-1131) |
| **China (n=1584)** |  |  |  |  |  |  |  |  |  |
| Daily total | 597 (349-961) | 107 (32-200) | 36 (0-129) | 193 (68-385) | 0 (0-50) | 0 (0-0) | 0 (0-43) | 0 (0-0) | 1214 (889-1684) |
| Meals | 77 (6-192) | 50 (0-114) | 0 (0-0) | 43 (0-136) | 0 (0-0) | 0 (0-0) | 0 (0-16) | 0 (0-0) | 325 (179-521) |
| Outside of meals | 0 (0-0) | 0 (0-0) | 0 (0-0) | 0 (0-0) | 0 (0-0) | 0 (0-0) | 0 (0-0) | 0 (0-0) | 0 (0-71) |
| Without food | 449 (249-750) | 26 (0-71) | 7 (0-86) | 100 (11-225) | 0 (0-0) | 0 (0-0) | 0 (0-0) | 0 (0-0) | 800 (518-1188) |
| **Indonesia (n=2778)** |  |  |  |  |  |  |  |  |  |
| Daily total | 2006 (1371-2824) | 0 (0-27) | 180 (0-391) | 85 (0-309) | 0 (0-0) | 0 (0-0) | ND | 0 (0-0) | 2599 (1827-3465) |
| Meals | 514 (257-823) | 0 (0-0) | 0 (0-54) | 0 (0-34) | 0 (0-0) | 0 (0-0) | ND | 0 (0-0) | 653 (400-950) |
| Outside of meals | 129 (0-380) | 0 (0-0) | 0 (0-106) | 0 (0-51) | 0 (0-0) | 0 (0-0) | ND | 0 (0-0) | 300 (65-607) |
| Without food | 1138 (636-1809) | 0 (0-0) | 34 (0-171) | 0 (0-153) | 0 (0-0) | 0 (0-0) | ND | 0 (0-0) | 1441 (858-2160) |

*SSB* sugared sweetened beverages, *FJ* fruit juices, *A/NSB* artificial/non-nutritive sweeteners beverages, *TFI* total fluid intake

Data presented as median (25, 75^th^ percentiles)

**Table S4** Median (P25-P75) fluid intake (mL/day) by location, by country and age group

| **Country** | **At home** | **At school/univ/work** | **Other locations** |
| --- | --- | --- | --- |
| **4-9 years** | | | |
| Mexico (n=293) | 931 (674-1283) | 0 (0-136) | 0 (0-34) |
| Brazil (n=146) | 1050 (763-1337) | 83 (0-199) | 72 (0-183) |
| Argentina (n=173) | 1404 (931-1935) | 169 (34-346) | 0 (0-188) |
| Uruguay (n=121) | 1400 (1152-1679) | 146 (104-284) | 0 (0-0) |
| China (n=279) | 451 (308-648) | 207 (103-364) | 110 (30-236) |
| Indonesia (n=388) | 1835 (1281-2687) | 0 (0-217) | 0 (0-0) |
| **10-17 years** | | | |
| Mexico (n=376) | 1139 (809-1670) | 23 (0-274) | 0 (0-155) |
| Brazil (n=194) | 1147 (779-1758) | 53 (0-180) | 101 (0-259) |
| Argentina (n=219) | 1497 (1025-1975) | 136 (0-357) | 21 (0-179) |
| Uruguay (n=144) | 1292 (885-1735) | 120 (0-235) | 0 (0-0) |
| China (n=370) | 403 (207-641) | 418 (241-631) | 143 (36-292) |
| Indonesia (n=478) | 1978 (1405-2678) | 109 (0-456) | 0 (0-34) |
| **≥ 18 years** | | | |
| Mexico (n=1677) | 1227 (832-1784) | 0 (0-171) | 0 (0-163) |
| Brazil (n=477) | 1168 (821-1735) | 0 (0-233) | 128 (12-328) |
| Argentina (n=1089) | 1593 (1079-2135) | 158 (0-579) | 36 (0-286) |
| Uruguay (n=554) | 1472 (884-2078) | 22 (0-516) | 0 (0-100) |
| China (n=1584) | 500 (279-767) | 361 (179-633) | 244 (89-492) |
| Indonesia (n=2778) | 2191 (1513-3018) | 0 (0-405) | 0 (0-43) |

*P25* 25^th^ percentiles, *P75* 75^th^ percentiles

**Table S5a** Mean (SEM) intake of different fluid types (mL/day) according to drinking locations among children (4-9 years), by country

| Country | Occasions | Water | | Milk & derivatives | | Hot beverages | | SSB | | 100% FJ | | A/NSB | | Alcoholic beverages | | Other beverages | | TFI | |
| --- | --- | --- | --- | --- | --- | --- | --- | --- | --- | --- | --- | --- | --- | --- | --- | --- | --- | --- | --- |
| **Mexico (n=293)** | Daily total | 384 | ±26 | 315 | ±16 | 37 | ±6 | 458 | ±23 | 19 | ±3 | 11 | ±3 | 0 | ±0 | 7 | ±3 | 1232 | ±39 |
|  | Home | 314 | ±22 | 306 | ±16 | 36 | ±6 | 392 | ±21 | 18 | ±3 | 10 | ±3 | 0 | ±0 | 6 | ±3 | 1083 | ±35 |
|  | School/univ/work | 58 | ±10 | 6 | ±2 | 1 | ±0 | 38 | ±5 | 0 | ±0 | 1 | ±1 | 0 | ±0 | 1 | ±1 | 105 | ±12 |
|  | Other locations | 13 | ±3 | 2 | ±1 | 1 | ±0 | 28 | ±5 | 0 | ±0 | 0 | ±0 | 0 | ±0 | 0 | ±0 | 44 | ±6 |
| **Brazil (n=146)** | Daily total | 455 | ±26 | 336 | ±21 | 44 | ±6 | 434 | ±25 | 120 | ±22 | 9 | ±3 | 0 | ±0 | 15 | ±4 | 1414 | ±56 |
|  | Home | 361 | ±21 | 295 | ±20 | 39 | ±6 | 325 | ±20 | 92 | ±19 | 8 | ±3 | 0 | ±0 | 10 | ±3 | 1131 | ±47 |
|  | School/univ/work | 56 | ±8 | 21 | ±4 | 2 | ±2 | 35 | ±6 | 16 | ±4 | 0 | ±0 | 0 | ±0 | 3 | ±2 | 133 | ±15 |
|  | Other locations | 37 | ±9 | 19 | ±4 | 2 | ±1 | 68 | ±9 | 11 | ±3 | 1 | ±0 | 0 | ±0 | 1 | ±1 | 140 | ±20 |
|  | Unspecified | 2 | ±1 | 0 | ±0 | 0 | ±0 | 6 | ±2 | 1 | ±0 | 0 | ±0 | 0 | ±0 | 1 | ±0 | 10 | ±2 |
| **Argentina (n=173)** | Daily total | 435 | ±41 | 364 | ±20 | 141 | ±14 | 711 | ±46 | 19 | ±5 | 133 | ±20 | 1 | ±0 | 2 | ±1 | 1807 | ±61 |
|  | Home | 338 | ±33 | 333 | ±19 | 111 | ±12 | 552 | ±38 | 16 | ±4 | 107 | ±17 | 1 | ±0 | 2 | ±1 | 1460 | ±50 |
|  | School/univ/work | 82 | ±13 | 17 | ±4 | 26 | ±5 | 85 | ±12 | 2 | ±1 | 16 | ±4 | 0 | ±0 | 0 | ±0 | 227 | ±19 |
|  | Other locations | 15 | ±5 | 14 | ±3 | 4 | ±1 | 75 | ±11 | 1 | ±1 | 10 | ±3 | 0 | ±0 | 0 | ±0 | 119 | ±15 |
| **Uruguay (n=121)** | Daily total | 535 | ±34 | 418 | ±28 | 1 | ±1 | 687 | ±44 | 4 | ±4 | 8 | ±5 | 0 | ±0 | 40 | ±13 | 1693 | ±52 |
|  | Home | 458 | ±32 | 384 | ±24 | 0 | ±0 | 587 | ±40 | 4 | ±4 | 6 | ±5 | 0 | ±0 | 21 | ±8 | 1461 | ±49 |
|  | School/univ/work | 72 | ±13 | 34 | ±9 | 1 | ±1 | 99 | ±21 | 0 | ±0 | 2 | ±1 | 0 | ±0 | 19 | ±8 | 226 | ±25 |
|  | Other locations | 5 | ±3 | 0 | ±0 | 0 | ±0 | 1 | ±1 | 0 | ±0 | 0 | ±0 | 0 | ±0 | 0 | ±0 | 6 | ±3 |
| **China (n=279)** | Daily total | 494 | ±21 | 269 | ±12 | 11 | ±2 | 128 | ±12 | 61 | ±7 | 1 | ±0 | 0 | ±0 | 2 | ±1 | 966 | 30 |
|  | Home | 260 | ±15 | 198 | ±11 | 2 | ±1 | 37 | ±4 | 25 | ±3 | 0 | ±0 | 0 | ±0 | 1 | ±1 | 524 | ±19 |
|  | School/univ/work | 174 | ±11 | 33 | ±3 | 3 | ±1 | 31 | ±4 | 17 | ±3 | 0 | ±0 | 0 | ±0 | 0 | ±0 | 260 | ±14 |
|  | Other locations | 59 | ±6 | 37 | ±3 | 6 | ±1 | 60 | ±8 | 19 | ±3 | 1 | ±0 | 0 | ±0 | 0 | ±0 | 182 | ±15 |
| **Indonesia (n=388)** | Daily total | 1663 | ±42 | 254 | ±16 | 68 | ±9 | 167 | ±12 | 6 | ±2 | 2 | ±1 | 0 | ±0 | 5 | ±2 | 2165 | ±45 |
|  | Home | 1553 | ±41 | 237 | ±16 | 62 | ±8 | 115 | ±9 | 6 | ±2 | 1 | ±1 | 0 | ±0 | 3 | ±1 | 1977 | ±44 |
|  | School/univ/work | 101 | ±11 | 15 | ±3 | 5 | ±2 | 38 | ±6 | 0 | ±0 | 0 | ±0 | 0 | ±0 | 0 | ±0 | 159 | ±14 |
|  | Other locations | 9 | ±2 | 2 | ±1 | 0 | ±0 | 15 | ±2 | 0 | ±0 | 1 | ±0 | 0 | ±0 | 2 | ±2 | 29 | ±4 |

*SEM* Standard error of the mean, *SSB* sugar sweetened beverages, *FJ* fruit juices, *A/NSB* artificial/non-nutritive sweeteners beverages, *TFI* total fluid intake

**Table S5b** Mean (SEM) intake of different fluid types (mL/day) according to drinking locations among adolescents (10-17 years), by country

| **Country** | Occasions | Water | | Milk & derivatives | | Hot beverages | | SSB | | 100% FJ | | A/NSB | | Alcoholic beverages | | Other beverages | | TFI | |
| --- | --- | --- | --- | --- | --- | --- | --- | --- | --- | --- | --- | --- | --- | --- | --- | --- | --- | --- | --- |
| **Mexico (n=376)** | Daily total | 639 | ±35 | 211 | ±12 | 102 | ±10 | 667 | ±29 | 25 | ±4 | 15 | ±3 | 4 | ±2 | 15 | ±4 | 1679 | ±47 |
|  | Home | 500 | ±29 | 194 | ±11 | 95 | ±10 | 506 | ±26 | 17 | ±3 | 11 | ±2 | 1 | ±0 | 9 | ±3 | 1333 | ±40 |
|  | School/univ/work | 100 | ±13 | 9 | ±2 | 3 | ±1 | 82 | ±9 | 3 | ±1 | 1 | ±1 | 0 | ±0 | 3 | ±1 | 200 | ±19 |
|  | Other locations | 39 | ±7 | 8 | ±2 | 5 | ±1 | 79 | ±10 | 6 | ±2 | 3 | ±2 | 3 | ±2 | 3 | ±2 | 146 | ±14 |
| **Brazil (n=194)** | Daily total | 630 | ±41 | 207 | ±17 | 102 | ±11 | 600 | ±32 | 121 | ±17 | 28 | ±5 | 21 | ±7 | 5 | ±2 | 1713 | ±69 |
|  | Home | 495 | ±30 | 176 | ±15 | 89 | ±10 | 458 | ±26 | 95 | ±16 | 21 | ±4 | 2 | ±1 | 2 | ±1 | 1339 | ±54 |
|  | School/univ/work | 56 | ±8 | 10 | ±2 | 7 | ±6 | 44 | ±7 | 11 | ±3 | 2 | ±1 | 0 | ±0 | 1 | ±1 | 131 | ±16 |
|  | Other locations | 73 | ±25 | 20 | ±5 | 5 | ±1 | 93 | ±10 | 13 | ±3 | 5 | ±1 | 18 | ±6 | 1 | ±1 | 229 | ±32 |
|  | Unspecified | 5 | ±2 | 1 | ±1 | 1 | ±1 | 5 | ±2 | 2 | ±1 | 0 | ±0 | 0 | ±0 | 0 | ±0 | 15 | ±3 |
| **Argentina (n=219)** | Daily total | 454 | ±31 | 222 | ±17 | 239 | ±19 | 818 | ±42 | 17 | ±5 | 128 | ±19 | 17 | ±5 | 2 | ±1 | 1897 | ±51 |
|  | Home | 356 | ±29 | 208 | ±17 | 211 | ±16 | 654 | ±38 | 14 | ±4 | 116 | ±18 | 5 | ±2 | 2 | ±1 | 1566 | ±47 |
|  | School/univ/work | 80 | ±10 | 10 | ±3 | 18 | ±5 | 96 | ±12 | 1 | ±0 | 8 | ±2 | 2 | ±2 | 0 | ±0 | 214 | ±18 |
|  | Other locations | 17 | ±5 | 4 | ±1 | 10 | ±3 | 68 | ±8 | 2 | ±1 | 4 | ±2 | 10 | ±4 | 0 | ±0 | 116 | ±12 |
| **Uruguay (n=144)** | Daily total | 561 | ±51 | 318 | ±26 | 164 | ±31 | 521 | ±41 | 21 | ±9 | 57 | ±14 | 2 | ±1 | 25 | ±6 | 1668 | ±68 |
|  | Home | 469 | ±44 | 278 | ±25 | 145 | ±29 | 401 | ±34 | 18 | ±8 | 47 | ±13 | 2 | ±1 | 18 | ±5 | 1379 | ±59 |
|  | School/univ/work | 60 | ±14 | 28 | ±7 | 10 | ±8 | 84 | ±17 | 3 | ±3 | 2 | ±2 | 0 | ±0 | 5 | ±2 | 192 | ±24 |
|  | Other locations | 31 | ±27 | 12 | ±5 | 9 | ±8 | 36 | ±13 | 0 | ±0 | 7 | ±4 | 0 | ±0 | 2 | ±1 | 97 | ±41 |
| **China (n=370)** | Daily total | 632 | ±27 | 189 | ±8 | 37 | ±6 | 253 | ±13 | 45 | ±4 | 7 | ±1 | 13 | ±4 | 1 | ±1 | 1177 | ±31 |
|  | Home | 270 | ±16 | 126 | ±7 | 9 | ±3 | 46 | ±4 | 14 | ±2 | 1 | ±0 | 2 | ±1 | 1 | ±0 | 468 | ±19 |
|  | School/univ/work | 303 | ±16 | 37 | ±3 | 15 | ±3 | 111 | ±7 | 15 | ±2 | 4 | ±1 | 1 | ±0 | 1 | ±1 | 487 | ±19 |
|  | Other locations | 59 | ±5 | 26 | ±3 | 13 | ±2 | 96 | ±7 | 16 | ±2 | 2 | ±0 | 10 | ±3 | 0 | ±0 | 222 | ±14 |
| **Indonesia (n=478)** | Daily total | 1933 | ±43 | 136 | ±11 | 121 | ±9 | 282 | ±16 | 9 | ±2 | 3 | ±2 | 0 | ±0 | 4 | ±1 | 2488 | ±49 |
|  | Home | 1700 | ±40 | 119 | ±10 | 110 | ±8 | 172 | ±12 | 6 | ±2 | 1 | ±0 | 0 | ±0 | 3 | ±1 | 2110 | ±45 |
|  | School/univ/work | 210 | ±16 | 10 | ±2 | 10 | ±2 | 80 | ±7 | 2 | ±1 | 1 | ±1 | 0 | ±0 | 1 | ±0 | 313 | ±20 |
|  | Other locations | 23 | ±6 | 7 | ±2 | 1 | ±1 | 31 | ±4 | 1 | ±0 | 1 | ±1 | 0 | ±0 | 0 | ±0 | 65 | ±9 |

*SEM* Standard error of the mean, *SSB* sugar sweetened beverages, *FJ* fruit juices, *A/NSB* artificial/non-nutritive sweeteners beverages, *TFI* total fluid intake

**Table S5c** Mean (SEM) intake of different fluid types (mL/day) according to drinking locations among adults (≥18 years), by country

| Country | Occasions | Water | | Milk & derivatives | | Hot beverages | | SSB | | 100% FJ | | A/NSB | | Alcoholic beverages | | | Other beverages | | | TFI | | |
| --- | --- | --- | --- | --- | --- | --- | --- | --- | --- | --- | --- | --- | --- | --- | --- | --- | --- | --- | --- | --- | --- | --- |
| Mexico (n=1677) | Daily total | 673 | ±18 | 132 | ±5 | 210 | ±7 | 655 | ±13 | 35 | ±3 | 23 | ±2 | 22 | ±2 | 6 | | ±1 | 1754 | | ±24 |  |
|  | Home | 554 | ±16 | 119 | ±4 | 193 | ±6 | 504 | ±11 | 27 | ±2 | 15 | ±2 | 10 | ±1 | 4 | | ±1 | 1426 | | ±21 |  |
|  | School/univ/work | 78 | ±7 | 4 | ±1 | 10 | ±1 | 83 | ±5 | 3 | ±1 | 5 | ±1 | 1 | ±1 | 0 | | ±0 | 184 | | ±10 |  |
|  | Other locations | 41 | ±4 | 8 | ±1 | 7 | ±1 | 68 | ±4 | 6 | ±1 | 3 | ±1 | 11 | ±2 | 1 | | ±0 | 145 | | ±7 |  |
| Brazil (n=477) | Daily total | 756 | ±28 | 92 | ±7 | 199 | ±10 | 490 | ±19 | 110 | ±7 | 23 | ±4 | 142 | ±12 | 10 | | ±2 | 1822 | | ±46 |  |
|  | Home | 614 | ±24 | 77 | ±6 | 165 | ±9 | 359 | ±16 | 78 | ±6 | 14 | ±2 | 63 | ±7 | 5 | | ±1 | 1376 | | ±38 |  |
|  | School/univ/work | 85 | ±8 | 6 | ±2 | 20 | ±3 | 56 | ±7 | 13 | ±2 | 5 | ±1 | 3 | ±2 | 3 | | ±2 | 191 | | ±18 |  |
|  | Other locations | 53 | ±6 | 8 | ±1 | 13 | ±2 | 72 | ±6 | 17 | ±2 | 3 | ±1 | 73 | ±8 | 2 | | ±1 | 242 | | ±15 |  |
|  | Unspecified | 4 | ±1 | 0 | ±0 | 1 | ±0 | 3 | ±1 | 0 | ±0 | 0 | ±0 | 3 | ±1 | 0 | | ±0 | 12 | | ±2 |  |
| Argentina (n=1089) | Daily total | 581 | ±20 | 60 | ±4 | 827 | ±17 | 488 | ±16 | 22 | ±3 | 160 | ±10 | 128 | ±8 | 6 | | ±1 | 2272 | | ±31 |  |
|  | Home | 436 | ±16 | 49 | ±4 | 629 | ±15 | 353 | ±13 | 17 | ±2 | 128 | ±9 | 73 | ±6 | 5 | | ±1 | 1691 | | ±26 |  |
|  | School/univ/work | 116 | ±9 | 5 | ±1 | 155 | ±9 | 83 | ±6 | 3 | ±1 | 18 | ±3 | 1 | ±0 | 0 | | ±0 | 382 | | ±17 |  |
|  | Other locations | 29 | ±4 | 6 | ±1 | 42 | ±3 | 52 | ±4 | 2 | ±0 | 14 | ±3 | 53 | ±5 | 1 | | ±1 | 199 | | ±10 |  |
| Uruguay (n=554) | Daily total | 493 | ±22 | 83 | ±6 | 873 | ±35 | 359 | ±17 | 26 | ±4 | 103 | ±10 | 48 | ±6 | 14 | | ±3 | 1999 | | ±43 |  |
|  | Home | 393 | ±18 | 73 | ±6 | 677 | ±30 | 269 | ±14 | 21 | ±3 | 77 | ±8 | 37 | ±5 | 10 | | ±2 | 1557 | | ±37 |  |
|  | School/univ/work | 80 | ±9 | 7 | ±2 | 151 | ±17 | 67 | ±9 | 5 | ±2 | 13 | ±3 | 0 | ±0 | 4 | | ±2 | 327 | | ±23 |  |
|  | Other locations | 20 | ±5 | 3 | ±1 | 44 | ±8 | 23 | ±5 | 1 | ±0 | 13 | ±3 | 11 | ±2 | 0 | | ±0 | 114 | | ±11 |  |
| China (n=1584) | Daily total | 751 | ±16 | 132 | ±3 | 136 | ±7 | 261 | ±7 | 35 | ±2 | 7 | ±1 | 62 | ±4 | 2 | | ±0 | 1387 | | ±18 |  |
|  | Home | 394 | ±11 | 81 | ±3 | 32 | ±2 | 45 | ±2 | 11 | ±1 | 2 | ±1 | 22 | ±2 | 0 | | ±0 | 588 | | ±13 |  |
|  | School/univ/work | 249 | ±9 | 25 | ±1 | 77 | ±5 | 99 | ±4 | 10 | ±1 | 2 | ±0 | 3 | ±1 | 0 | | ±0 | 466 | | ±11 |  |
|  | Other locations | 108 | ±4 | 26 | ±1 | 27 | ±2 | 116 | ±4 | 14 | ±1 | 3 | ±0 | 37 | ±2 | 1 | | ±0 | 333 | | ±8 |  |
| **Indonesia (n=2778)** | Daily total | 2164 | ±20 | 52 | ±3 | 263 | ±6 | 227 | ±7 | 12 | ±1 | 2 | ±0 | 0 | ±0 | 2 | | ±0 | 2721 | | ±22 |  |
|  | Home | 1943 | ±20 | 46 | ±2 | 224 | ±5 | 138 | ±5 | 7 | ±1 | 1 | ±0 | 0 | ±0 | 1 | | ±0 | 2361 | | ±21 |  |
|  | School/univ/work | 185 | ±7 | 3 | ±0 | 32 | ±2 | 57 | ±3 | 2 | ±0 | 1 | ±0 | 0 | ±0 | 0 | | ±0 | 279 | | ±9 |  |
|  | Other locations | 35 | ±3 | 3 | ±0 | 8 | ±1 | 32 | ±2 | 2 | ±0 | 0 | ±0 | 0 | ±0 | 0 | | ±0 | 81 | | ±5 |  |

*SEM* Standard error of the mean, *SSB* sugar sweetened beverages, *FJ* fruit juices, *A/NSB* artificial/non-nutritive sweeteners beverages, *TFI* total fluid intake

**Fig S1a** Contribution of the different fluid types to total fluid intake according to drinking occasion of children (4-9y), by country

**Fig S1b** Contribution of the different fluid types to total fluid intake according to drinking occasion of adolescents (10-17 y), by country

**Fig S1c** Contribution of the different fluid types to total fluid intake according to drinking occasion of adults (≥18 y), by country

**Fig S2** Contribution of the fluid intake for each location to the total fluid intake (%), by country and age group

**Fig S3a** Contribution of the different fluid types to total fluid intake according to drinking occasion of children (4-9 years), by country

**Fig S3b** Contribution of the different fluid types to total fluid intake according to drinking occasion of children (10-17 years), by country

**Fig S3c** Contribution of the different fluid types to total fluid intake according to drinking occasion of adults (≥18 years), by country
